# Obstetric ICU: Analysing and Understanding the Data is Important

**DOI:** 10.5005/jp-journals-10071-23158

**Published:** 2019-05

**Authors:** Sunil T Pandya1, Shwetha Mogal, Atul P Kulkarni

**Affiliations:** 1 Department of Surgical and Obstetric Critical Care, Century Hospital, Hyderabad, Telangana, India; 2 Department of Anesthesia, Pain Medicine and Obstetric Critical Care, Hyderabad, Telangana, India; 3 Division of Critical Care Medicine, Department of Anesthesiology, Critical Care and Pain, Tata Memorial Hospital, Homi Bhabha National Institute, Mumbai, Maharashtra, India

## Abstract

**How to cite this article:** Pandya ST, Mogal S, Kulkarni AP. Obstetric ICU: Analysing and Understanding the Data is Important. Indian J Crit Care Med 2019;23(5):201–202.

Maternal mortality remains a significant public health problem worldwide despite advances in obstetrics and healthcare for pregnant women. Maternal mortality shows a differential rate between countries and within countries with higher rates prevalent among the underprivileged populations. Maternal mortality is rarely an isolated phenomenon and is often a combination of several factors including the health of the pregnant woman, healthcare resources, and the environment. These include, but are not limited to, maternal demographics and associated morbidity, access, availability and affordability of complete health care infrastructure and resources during pregnancy and postpartum, maternal nutrition and comorbidity, and interventions during pregnancy.

Pregnancy induced hypertension, maternal anemia and hemorrhage remain significant contributors to maternal morbidity and mortality. Several relatively newer contributors to maternal mortality have to be recognized. Advances in reproductive health, obstetrics, fetal medicine and neonatal care have led to a gradual increase in the proportion of older pregnant women. Women who delayed pregnancy for personal, career or medical reasons have options for childbirth that were not available earlier. There is an increasing trend towards higher maternal age at first pregnancy.^1,2^ As maternal age at pregnancy increases, the number of women in childbearing age presenting with severe comorbidities is also rising.^3,4^

Epidemiological transitions in public health have led to a shift from a communicable model of disease to an increasing incidence and prevalence of noncommunicable diseases at younger ages. Chronic diseases like diabetes mellitus, hypertension, cardiac diseases, obesity etc. are increasingly prevalent even in developing economies translating to an increasing number of pregnant women diagnosed with chronic hypretension, cardiac diseases, diabetes mellitus and gestational diabetes and maternal obesity. The effects of reproductive transitions on women's health is also increasingly recognized.^5^ The life course approach to women's health recognizes that health outcomes in women are shaped by biological and social factors that act interactively and cumulatively throughout the entire lifespan, including puberty and premenopausal periods.

We also have to consider the long-term effects of modern and innovative medical treatments on young girls and children of women who have conceived using artificial reproductive techniques and who have now reached an age where they can conceive.^6^ As a consequence, pregnancies of women with pre-existing conditions are increasing and leading to an increased risk for peripartum complications^7^ and enhanced multidisciplinary care.^8^

Recent achievements of perinatal medicine including obstetric critical care, fetal and neonatal medicine, and improvements in addressing maternal illness have led to an increased confidence to use interventions that aid maternal and fetal survival. Improved diagnostic metrics and the development and use of different scoring systems in both resource limited and advanced nations^9^ that address specific components of critical care have led to the possibility of early intervention and improved probability of survival. However, this has also led to a steady high rate of preterm delivery as well as to direct and indirect short and long term maternal morbidity.

These challenges have led to an increasing utilization of intensive care unit services for pregnant women where women can be managed by a multidisciplinary team. In this issue of the journal, Vargas and colleagues have reported a wide variation in the prevalence of ICU admissions among obstetric patients ranging from 1 to 9 per 1000 pregnancies.^10,11^ Vargas and colleagues have reported high incidence of previous cesarean section among women admitted to the ICU, requiring peripartum hysterectomy. They have also reported significantly higher injury severity scores at ICU admission in developing nations compared to that of developed countries, thus emphasizing delay in accessing ICU care is the leading factor of maternal mortality. They have also reported that the most frequent cause of ICU admission in obstetric group is major hemorrhages (31%) followed by hypertensive disorders (25%). Hemodynamic instability is also a major reason for ICU admissions. In the United Kingdom and United States, the ICU admission rate is 0.9% of all pregnancies, with mortality rates ranging from 5 to 20%. The wide variation in admission rates may possibly reflect the variation in diagnostic criteria, criteria for admission to an ICU and variations in healthcare infrastructure that can identify and manage a condition before it progresses to a level that requires care in an ICU.

Increasing the number of ICUs and resources in ICUs to deal with pregnant women may be one solution to address maternal mortality and severe maternal morbidity. However, considering advances in noninvasive and invasive procedures including advances in providing organ supports in a critically ill parturient with multi-organ failure (e.g. VV ECMO, VA ECMO, LVADs, RRTs, radiological interventions in hybrid OT, bridging to organ transplantation etc),^12^ it might be more pragmatic to plan for an intermediate level of advanced care through a dedicated obstetric ICU model. This will allow ICU resources to be used for those who require invasive procedures or the most needy. The obstetric ICU through an integrated management model involving intensivists, obstetricians and anesthetists can take care of those who need more intense monitoring but can be managed through noninvasive methods.

The future of obstetrics may possibly be its evolution into a multidisciplinary team taking care of the pregnant women from preconception through the postpartum period. Such an integrated approach may help identify morbidity early, manage existing and newly identified conditions during pregnancy, plan strategies for childbirth including time, mode, and post childbirth care and provide advice for interpregnancy care. Such an approach may reduce the admission rates to an ICU and improve maternal mortality rates. In the meantime, it is imperative that ICUs with obstetric patients work to develop common parameters for ICU admissions, standardize protocols for diagnosis and management, document and analyse data for trends, risk factors and outcomes.
